# Association of Postoperative Antidepressant and Anxiolytic Exposure With Overall Survival Following Radical Cystectomy for Bladder Cancer

**DOI:** 10.1002/pon.70581

**Published:** 2026-08-17

**Authors:** Cheng‐Shuo Huang, Kai‐Hsiang Tung, Ying‐Si Wu, Cheng‐Ping Yu, Dah‐Shyong Yu, Ming‐Hsin Yang, Yong‐Eng Lee, Jar‐Yi Ho

**Affiliations:** ^1^ College of Biomedical Sciences Graduate Institute of Life Sciences National Defense Medical University Taipei Taiwan; ^2^ College of Medicine Graduate Institute of Pathology and Parasitology National Defense Medical University Taipei Taiwan; ^3^ Department of Pathology Tri‐Service General Hospital National Defense Medical University Taipei Taiwan; ^4^ National Institute of Cancer Research National Health Research Institutes Miaoli Taiwan; ^5^ Department of Integrative Biology and Pharmacology McGovern Medical School the University of Texas Health Science Center at Houston Houston Texas USA; ^6^ Department of Surgery Division of Urology Tri‐Service General Hospital National Defense Medical University Taipei Taiwan; ^7^ Department of Psychiatry Tri‐Service General Hospital Beitou Branch Taipei Taiwan; ^8^ Department of Orthopedic Surgery Shin Kong Wu Ho‐Su Memorial Hospital Taipei Taiwan

**Keywords:** anti‐anxiety agents, antidepressive agents, bladder neoplasms, cancer, cystectomy, mental health, mortality, oncology, survival analysis, survivorship

## Abstract

**Background:**

Psychological distress is an important component of cancer survivorship, particularly among patients undergoing radical cystectomy (RC) for bladder cancer. However, the prognostic significance of postoperative psychiatric medication exposure following RC remains unclear.

**Aims:**

To investigate the association between postoperative antidepressant and/or anxiolytic exposure and survival outcomes following RC for bladder cancer.

**Methods:**

This retrospective propensity score–matched cohort study used data from the TriNetX US Collaborative Network. Adult patients who underwent RC for bladder cancer (2005–2025) were identified. Postoperative psychiatric medication exposure was defined as new use of antidepressants (Anatomical Therapeutic Chemical: N06A) and/or anxiolytics (ATC: N05B). The primary analysis evaluated combined antidepressant/anxiolytic exposure, with separate antidepressant‐only and anxiolytic‐only subgroup analyses. Overall survival (OS) and all‐cause mortality were evaluated after 1:1 propensity score matching (PSM).

**Results:**

The primary analysis identified 2461 exposed and 7257 unexposed patients, yielding 1521 matched pairs after PSM. Patients exposed to postoperative psychiatric medication were associated with significantly poorer OS than their matched controls (hazard ratio [HR]: 1.377, 95% confidence interval [CI]: 1.261–1.502; log‐rank *p* < 0.001). Similar associations were observed in subgroup analyses. Antidepressant‐only exposure was associated with poorer OS (HR: 1.260, 95% CI: 1.161–1.367; *p* < 0.001), and the anxiolytic‐only subgroup demonstrated the strongest association with poorer survival (HR: 1.457, 95% CI: 1.324–1.604; *p* < 0.001).

**Conclusions:**

Psychiatric medication use may represent a clinically observable marker of psychological burden and survivorship vulnerability following RC. Early recognition of postoperative psychological vulnerability may facilitate timely psycho‐oncological support and multidisciplinary survivorship care following RC.

## 1 Background

Bladder cancer is among the most common malignancies of the urinary tract and remains an important global health burden. In the United States, approximately 84,530 new cases and 17,870 deaths from bladder cancer are projected in 2026 (National Cancer Institute. Cancer Stat Facts: Bladder Cancer. Surveillance, Epidemiology, and End Results Programme (SEER). Accessed 24 May 2026. https://seer.cancer.gov/statfacts/html/urinb.html). Approximately 25%–30% of patients present with muscle‐invasive bladder cancer (MIBC), a biologically aggressive disease associated with a high risk of recurrence, metastasis and cancer‐related mortality. Radical cystectomy (RC) with pelvic lymph node dissection remains the standard curative treatment for eligible patients with localised MIBC and selected cases of high‐risk bladder cancer. Despite aggressive multimodal management, MIBC continues to carry a substantial risk of recurrence and cancer‐related mortality [1, 2, 3]. Contemporary studies report 5‐year post‐cystectomy survival rates of approximately 50%–60%, while patients with locally advanced or extravesical disease remain particularly vulnerable to disease recurrence and treatment failure [4].

Although RC provides effective oncological control, the postoperative survivorship burden remains substantial. In addition to the challenges of cancer treatment, patients frequently experience significant physiological, functional and psychosocial sequelae following surgery. Urinary diversion, impaired body image, sexual dysfunction, chronic fatigue, altered daily functioning and difficulties with social reintegration markedly diminish long‐term quality of life [5, 6]. Consequently, the management of MIBC has evolved beyond a sole focus on oncological control to include survivorship‐related outcomes, particularly postoperative functional recovery, psychosocial adaptation and overall well‐being [7].

Psychological distress is increasingly recognised as an important yet frequently under‐addressed aspect of cancer survivorship, particularly among patients undergoing intensive treatments such as RC for MIBC. Following surgery, many patients experience persistent anxiety, depressive symptoms, fear of recurrence, social withdrawal and difficulties adapting to urinary diversion, altered body image and treatment‐related functional limitations. The consequences of these psychological challenges extend beyond emotional well‐being and may adversely affect treatment adherence, healthcare utilisation, rehabilitation, sleep quality, nutritional status and overall recovery [8, 9, 10]. From a psycho‐oncology perspective, the identification and management of common mental disorders are integral to comprehensive survivorship care. Untreated psychiatric comorbidities have been associated with greater disability, poorer quality of life and progressive functional decline. Meta‐analytic evidence suggests that approximately one‐third of cancer patients in acute care settings meet criteria for a clinically relevant mental health disorder, highlighting a substantial unmet need for psychosocial assessment and intervention in oncology practice [11, 12]. In bladder cancer, population‐based studies have reported a high incidence of psychiatric morbidity following treatment for MIBC, with psychiatric disorders being associated with worse overall and cancer‐specific survival [13]. These observations suggest that postoperative depression and anxiety may represent clinically meaningful markers of survivorship vulnerability and unmet supportive care needs following RC. However, psychological distress is frequently under‐recognised, under‐assessed and incompletely documented in routine oncology care and electronic health records, limiting opportunities to systematically examine its long‐term prognostic implications and motivating investigation of pragmatic real‐world clinical markers [14].

**FIGURE 1:**
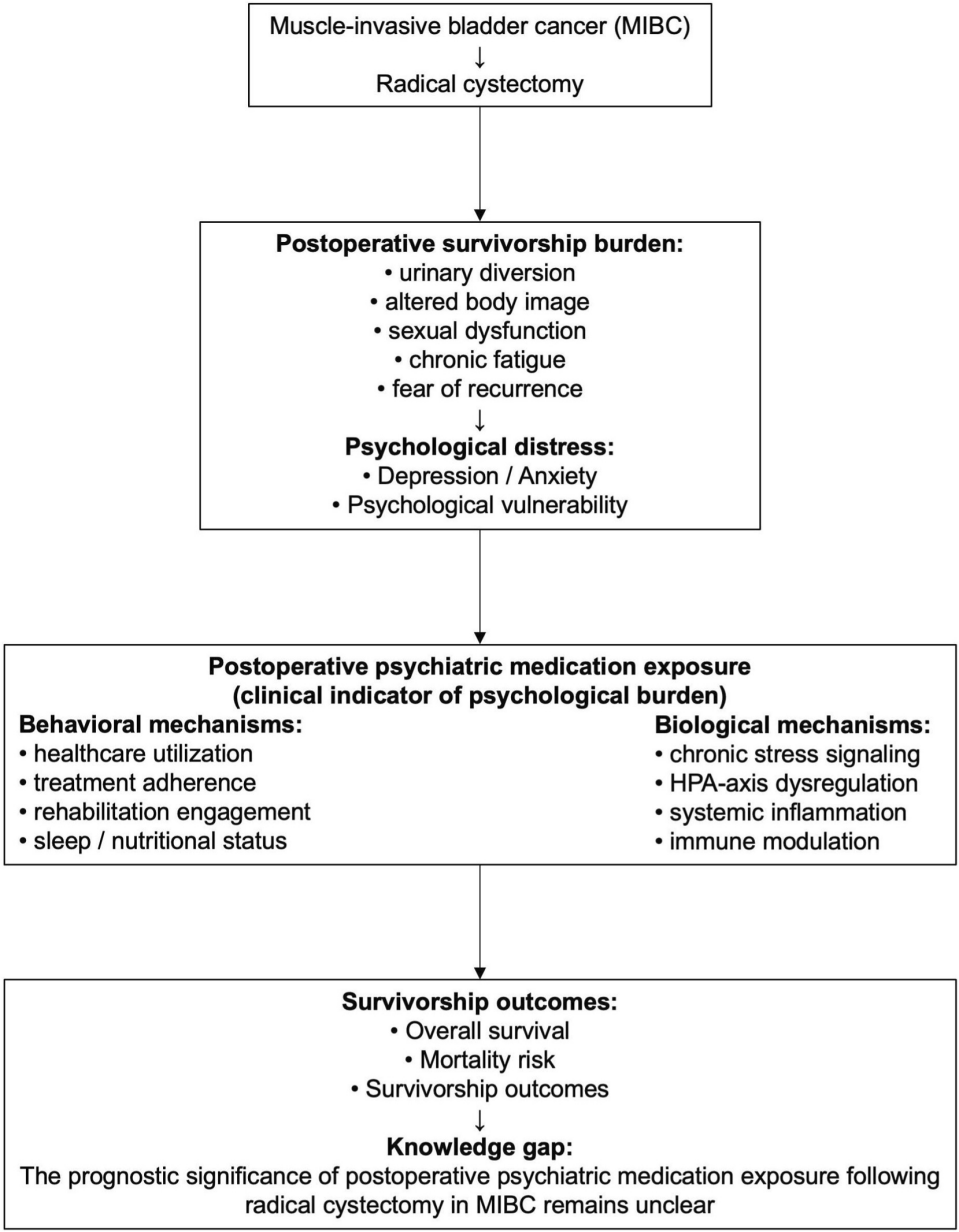
Conceptual framework linking postoperative psychiatric medication exposure to survivorship outcomes following radical cystectomy for muscle‐invasive bladder cancer. Radical cystectomy may impose substantial postoperative survivorship burden, including functional, psychosocial and quality‐of‐life challenges. Postoperative exposure to psychiatric medication may serve as a clinically observable marker of psychological vulnerability and may plausibly influence survivorship outcomes through behavioural and biological pathways. The prognostic significance of postoperative psychiatric medication exposure following radical cystectomy remains incompletely understood, providing the clinical rationale for the present study.

Importantly, postoperative psychiatric medication exposure may have implications beyond symptom control and may serve as a clinically observable marker of psychological vulnerability following RC. Emerging evidence suggests complex interactions among psychiatric disorders, psychotropic medication use and oncological outcomes, mediated through mechanisms such as chronic stress signalling, hypothalamic–pituitary–adrenal (HPA) axis dysregulation, systemic inflammation, immune modulation, behavioural factors and healthcare utilisation patterns [15, 16]. Within the broader framework of psychoneuroimmunology and cancer survivorship, these pathways provide biological and clinical plausibility for an association between postoperative psychiatric medication exposure and long‐term oncological outcomes [17, 18]. A conceptual framework summarising these hypothesised relationships is presented in Figure 1.

Despite growing interest in the relationship between psychological health and cancer outcomes, the available evidence remains limited, heterogeneous and largely derived from broad oncology populations rather than disease‐specific surgical cohorts. Although psychological distress is increasingly recognised as an important component of bladder cancer survivorship, the prognostic significance of postoperative psychiatric medication exposure following RC remains poorly defined. In particular, it remains unclear whether postoperative initiation of antidepressants or anxiolytics is associated with long‐term oncological outcomes or may serve as a pragmatic real‐world marker of postoperative psychological burden among patients with MIBC [19]. To address this knowledge gap, we conducted a retrospective propensity score–matched cohort study using the TriNetX federated research network to investigate the association between postoperative psychiatric medication exposure and survivorship outcomes following RC for MIBC. Specifically, we examined whether postoperative exposure to antidepressants and/or anxiolytics was associated with overall survival (OS) and all‐cause mortality.

## 2 Methods

### 2.1 Data Source and Study Design

A retrospective cohort study was conducted using the TriNetX US Collaborative Network, a federated real‐world database that aggregates de‐identified electronic health records from multiple healthcare organisations across the United States. Data were queried in May 2026 using the TriNetX Analytics Platform through institutional access provided by Shin Kong Wu Ho‐Su Memorial Hospital. The database contains longitudinal clinical information including demographic characteristics, diagnoses, procedures, medication records and survival outcomes. The study was approved by the Institutional Review Board of Tri‐Service General Hospital (Approval No. A202505107 and B202605014). The requirement for informed consent was waived owing to the retrospective study design. As TriNetX provides only aggregated and de‐identified patient data, investigators had no access to directly identifiable patient information.

### 2.2 Study Population and Cohort Definition

Eligible patients were adults (≥ 18 years) with a diagnosis of bladder cancer (ICD‐10‐CM code: C67) recorded between 1 January 2005 and 1 January 2025 who subsequently underwent RC. RC was identified using ICD‐10‐PCS procedure codes (0TTB0ZZ and 0TTB4ZZ) and (Current Procedural Terminology) codes (51570 and 51575), with robotic‐assisted procedures included where applicable. Given the indications for surgery, the study population was considered representative of patients undergoing RC for predominantly MIBC or other high‐risk bladder cancer. To minimise potential confounding by pre‐existing psychiatric treatment, patients with documented exposure to antidepressant or anxiolytic medications on or before the date of their first RC were excluded from the exposed cohort. This restriction allowed the analysis to focus on psychiatric medication use initiated after surgery.

### 2.3 Exposure Definition

The exposure of interest was postoperative psychiatric medication use, defined as the initiation of antidepressant and/or anxiolytic therapy after RC. Antidepressant exposure was identified using the Anatomical Therapeutic Chemical (ATC) classification code N06A, and anxiolytic exposure using ATC code N05B. To increase the likelihood of clinically meaningful medication exposure and minimise isolated or incidental prescriptions, exposed patients were required to have at least three prescription records for the relevant medication class documented in the electronic health record after surgery. This criterion was intended to reduce potential misclassification arising from short‐term perioperative prescribing or sporadic medication use. The requirement for at least three postoperative prescriptions was proposed to improve exposure specificity by reducing misclassification arising from isolated or transient prescribing. This would help better identify patients receiving sustained postoperative psychiatric pharmacotherapy.

Two cohorts were defined:

Exposed cohort: patients who initiated antidepressants and/or anxiolytics after RC and had at least three documented prescription records.

Non‐exposed cohort: patients with no recorded antidepressant or anxiolytic exposure during the study period.

### 2.4 Outcome Measures

The primary outcome was OS, assessed using the TriNetX mortality endpoint, defined by documented deceased status within the database. OS was evaluated using Kaplan–Meier survival analysis and Cox proportional hazards models within the TriNetX platform. Kaplan–Meier methods were used to estimate cumulative survival probabilities, differences between cohorts were assessed using the log‐rank test, and Cox proportional hazards models were used to estimate hazard ratios (HRs) and corresponding 95% confidence intervals (CIs) for the association between postoperative psychiatric medication exposure and OS. Numbers and proportions of deaths were summarised descriptively for each matched cohort.

### 2.5 Propensity Score Matching and Statistical Analysis

To minimise baseline differences between the exposure groups, 1:1 propensity score matching (PSM) was performed using the built‐in matching algorithm available within the TriNetX platform. Covariates included in the matching process comprised demographic characteristics (age, sex, race and ethnicity), major comorbidities (hypertension, diabetes mellitus, chronic kidney disease, hyperlipidaemia, obesity, cardiovascular disorders, neurologic disorders and mental and behavioural disorders), prior procedural history, medication exposure categories and oncology‐related variables when available (tumour stage, TNM classification and bladder cancer histology‐related coding variables). Covariate balance before and after matching was evaluated using standardised mean differences (SMDs), with an SMD of < 0.1 considered indicative of adequate balance between cohorts. All statistical analyses were conducted within the TriNetX Analytics environment. Statistical tests were two‐sided, and a *p*‐value of < 0.05 was considered statistically significant.

## 3 Results

### 3.1 Cohort Selection and Baseline Characteristics

A total of 9718 adult patients who underwent RC for bladder cancer between 1 January 2005 and 1 January 2025 were identified from the TriNetX US Collaborative Network. In the primary analysis, 2461 patients met the criteria for postoperative psychiatric medication exposure, whereas 7257 patients had no documented postoperative psychiatric medication exposure. Following 1:1 PSM, 1521 matched pairs were available for the combined exposure analysis.

**FIGURE 2:**
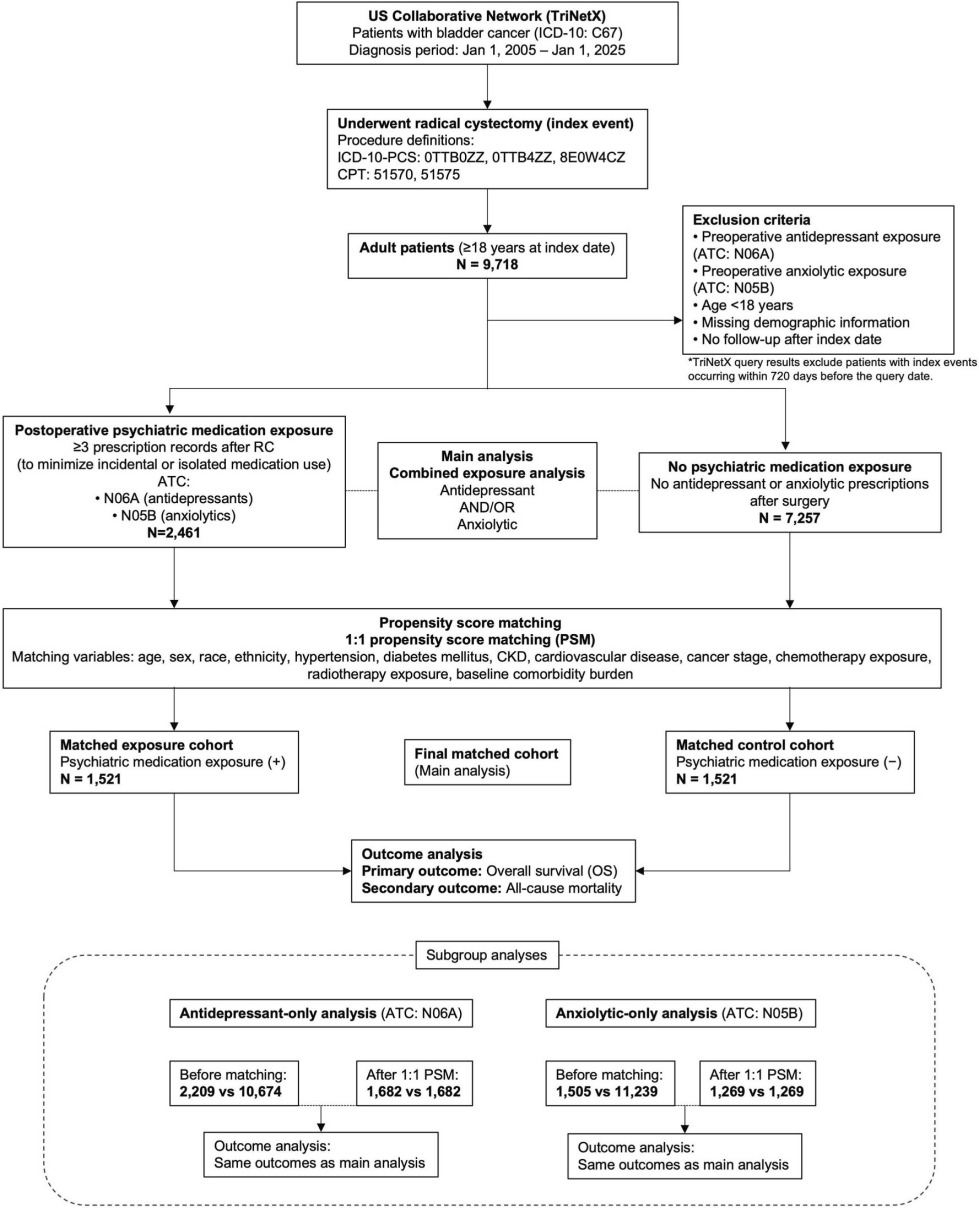
Cohort selection flowchart and study design. Flowchart illustrating patient selection, cohort construction, propensity score matching and subgroup analyses. Adult patients who underwent radical cystectomy for bladder cancer were identified from the TriNetX US Collaborative Network (2005–2025). Patients with documented antidepressant or anxiolytic exposure before radical cystectomy were excluded. Postoperative psychiatric medication exposure was defined as the initiation of antidepressants (ATC code N06A) and/or anxiolytics (ATC code N05B) after surgery, with at least 3 recorded prescriptions required to minimise misclassification arising from incidental or isolated medication use. The primary analysis evaluated combined antidepressant/anxiolytic exposure versus non‐exposure following 1:1 propensity score matching. Separate antidepressant‐only and anxiolytic‐only subgroup analyses were conducted using the same analytical framework. Overall survival and all‐cause mortality were evaluated as study outcomes. ATC, anatomical therapeutic chemical classification; CKD, chronic kidney disease; CVD, cardiovascular disease; DM, diabetes mellitus; OS, overall survival; PSM, propensity score matching.

Prespecified subgroup analyses were performed to evaluate the associations of antidepressant and anxiolytic use separately. In the antidepressant‐only analysis, 2209 exposed patients and 10,674 controls were identified before matching, yielding 1682 matched pairs after PSM. In the anxiolytic‐only analysis, 1505 exposed patients and 11,239 unexposed controls were identified before matching, resulting in 1269 matched pairs after PSM. The cohort selection process is summarised in Figure 2.

Before PSM, baseline differences were observed between the exposed and non‐exposed cohorts across demographic, clinical and tumour‐related variables. In the combined exposure analysis, patients who received postoperative psychiatric medications had higher prevalences of hypertension, chronic kidney disease, circulatory diseases, neurological disorders and mental/behavioural disorders than those without psychiatric medication exposure. Differences in racial composition and cancer stage were also present before matching. Following 1:1 PSM, baseline characteristics were well balanced, with SMDs < 0.1 across all matched variables (Table 1). Similar improvements in covariate balance were observed in the antidepressant‐only and anxiolytic‐only subgroup analyses.

**TABLE 1:** Baseline demographic, clinical and tumour characteristics of patients with and without postoperative antidepressant and/or anxiolytic exposure before and after propensity score matching.

Variable	Before PSM, *n* (%)			After PSM, *n* (%)		
	Combined exposure (+) (*N* = 2461)	Control (*N* = 7257)	Std Diff	Combined exposure (+) (*N* = 1521)	Control (*N* = 1521)	Std Diff
Demographics						
Age, mean ± SD	69.6 ± 10.4	69.4 ± 10.0	0.017	69.9 ± 10.5	70.0 ± 10.1	0.007
Female sex	621 (25.4%)	1530 (21.3%)	0.097	355 (23.3%)	353 (23.2%)	0.003
White race	2018 (82.6%)	5593 (77.9%)	0.119	1197 (78.7%)	1206 (79.3%)	0.015
Black/African american	169 (6.9%)	579 (8.1%)	0.044	113 (7.4%)	120 (7.9%)	0.017
Asian	101 (4.1%)	388 (5.4%)	0.06	84 (5.5%)	80 (5.3%)	0.012
Hispanic/latino ethnicity	78 (3.2%)	268 (3.7%)	0.03	52 (3.4%)	60 (3.9%)	0.028
Clinical comorbidities						
Hypertension	1746 (71.5%)	3627 (50.5%)	0.44	1052 (69.2%)	1061 (69.8%)	0.013
Hyperlipidaemia	1298 (53.2%)	2351 (32.8%)	0.421	775 (51.0%)	781 (51.3%)	0.008
Diabetes mellitus	700 (28.7%)	1414 (19.7%)	0.211	433 (28.5%)	432 (28.4%)	0.001
Chronic kidney disease (CKD)	913 (37.4%)	1158 (16.1%)	0.495	478 (31.4%)	475 (31.2%)	0.004
Obesity/overweight	638 (26.1%)	934 (13.0%)	0.335	369 (24.3%)	368 (24.2%)	0.002
Circulatory diseases	2163 (88.6%)	4821 (67.2%)	0.534	1309 (86.1%)	1307 (85.9%)	0.004
Genitourinary diseases	2269 (92.9%)	5200 (72.4%)	0.562	1387 (91.2%)	1398 (91.9%)	0.026
Nervous system disorders	1660 (68.0%)	1821 (25.4%)	0.944	832 (54.7%)	834 (54.8%)	0.003
Mental/behavioural disorders	1446 (59.2%)	1638 (22.8%)	0.796	667 (43.9%)	689 (45.3%)	0.029
Tumour characteristics						
Stage II disease	157 (6.4%)	269 (3.7%)	0.122	63 (4.1%)	59 (3.9%)	0.013
Stage III disease	110 (4.5%)	139 (1.9%)	0.146	43 (2.8%)	42 (2.8%)	0.004
Stage IV disease	59 (2.4%)	57 (0.8%)	0.129	21 (1.4%)	18 (1.2%)	0.018

### 3.2 Postoperative Psychiatric Medication Exposure Was Associated With Poorer Overall Survival

#### 3.2.1 Combined Exposure Analysis (Primary Analysis)

**FIGURE 3:**
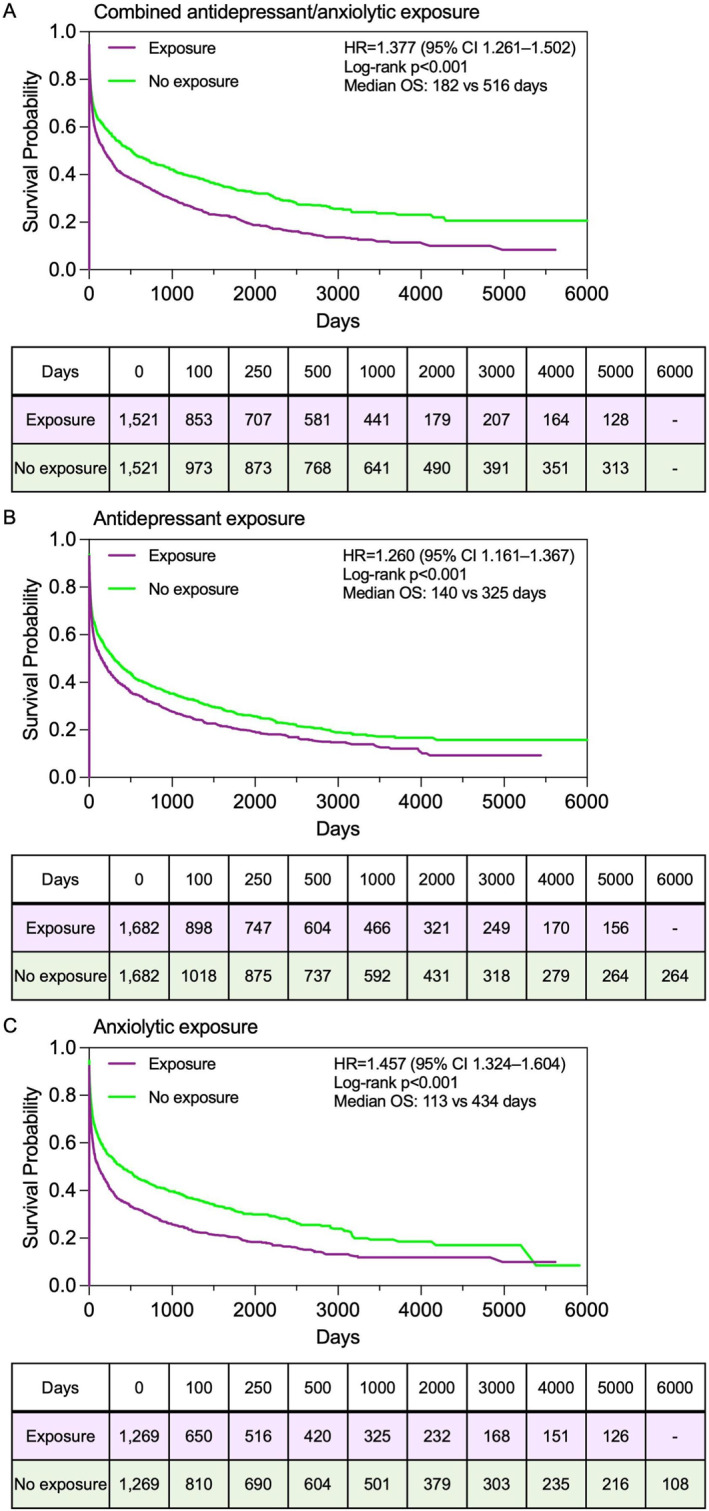
Kaplan–Meier analysis of overall survival according to postoperative psychiatric medication exposure after propensity score matching (A) Combined antidepressant/anxiolytic exposure cohort. (B) Antidepressant‐only cohort. (C) Anxiolytic‐only cohort. Patients with postoperative psychiatric medication exposure demonstrated significantly poorer overall survival compared with matched non‐exposed controls. Hazard ratios, 95% confidence intervals, log‐rank *p* values and median overall survival are shown in each panel.

In the primary combined exposure analysis, postoperative psychiatric medication exposure was associated with poorer OS after RC. Following 1:1 PSM, 1115 of the 1521 exposed patients died during follow‐up compared with 920 of the 1521 matched controls. Kaplan–Meier analysis demonstrated significantly shorter OS in the exposed cohort, with a median OS of 182 versus 516 days (HR: 1.377, 95% CI: 1.261–1.502; log‐rank *p* < 0.001) (Figure 3A, Table 2).

**TABLE 2:** Association between postoperative psychiatric medication exposure and survival outcomes after propensity score matching.

Analysis	Matched cohort (n)	Deaths, Exp	Deaths, Ctrl	Risk, Exp	Risk, Ctrl	RR (95% CI)	OR (95% CI)	Median OS, Exp versus Ctrl (days)	HR (95% CI)	Log‐rank *p*
Combined AD/AX exposure	1521 versus 1521	1115	920	0.733	0.605	1.212 (1.152–1.275)	1.794 (1.539–2.091)	182 versus 516	1.377 (1.261–1.502)	< 0.001
AD exposure only	1682 versus 1682	1218	1115	0.724	0.663	1.092 (1.044–1.143)	1.335 (1.152–1.546)	140 versus 325	1.260 (1.161–1.367)	< 0.001
AX exposure only	1269 versus 1269	909	785	0.716	0.619	1.158 (1.096–1.224)	1.557 (1.318–1.839)	113 versus 434	1.457 (1.324–1.604)	< 0.001

*Note:* Overall survival was assessed using Kaplan–Meier analysis after 1:1 propensity score matching.

Abbreviations: AD, antidepressant; AX, anxiolytic; CI, confidence interval; Ctrl, control; Exp, exposure; HR, hazard ratio; OR, odds ratio; OS, overall survival; RR, risk ratio.

#### 3.2.2 Antidepressant‐Only Subgroup Analysis

The association between postoperative psychiatric medication use and poorer survival remained evident in the antidepressant‐only subgroup analysis. Following 1:1 PSM, 1218 of the 1682 antidepressant‐exposed patients died during follow‐up compared with 1115 of the 1682 matched controls. Kaplan–Meier analysis demonstrated significantly poorer OS among antidepressant‐exposed patients, with a median OS of 140 versus 325 days (HR: 1.260, 95% CI: 1.161–1.367; log‐rank *p* < 0.001) (Figure 3B, Table 2).

#### 3.2.3 Anxiolytic‐Only Subgroup Analysis

The association between postoperative anxiolytic use and poorer OS remained evident in the anxiolytic‐only subgroup analysis. Following 1:1 PSM, 908 of the 1269 anxiolytic‐exposed patients died during follow‐up compared with 785 of the 1269 matched controls. Kaplan–Meier analysis demonstrated significantly poorer OS among patients who received postoperative anxiolytics, with a median OS of 113 versus 434 days (HR: 1.457, 95% CI: 1.324–1.604; log‐rank *p* < 0.001) (Figure 3C, Table 2).

#### 3.2.4 Integrated Comparison Across Exposure Analyses

**FIGURE 4:**
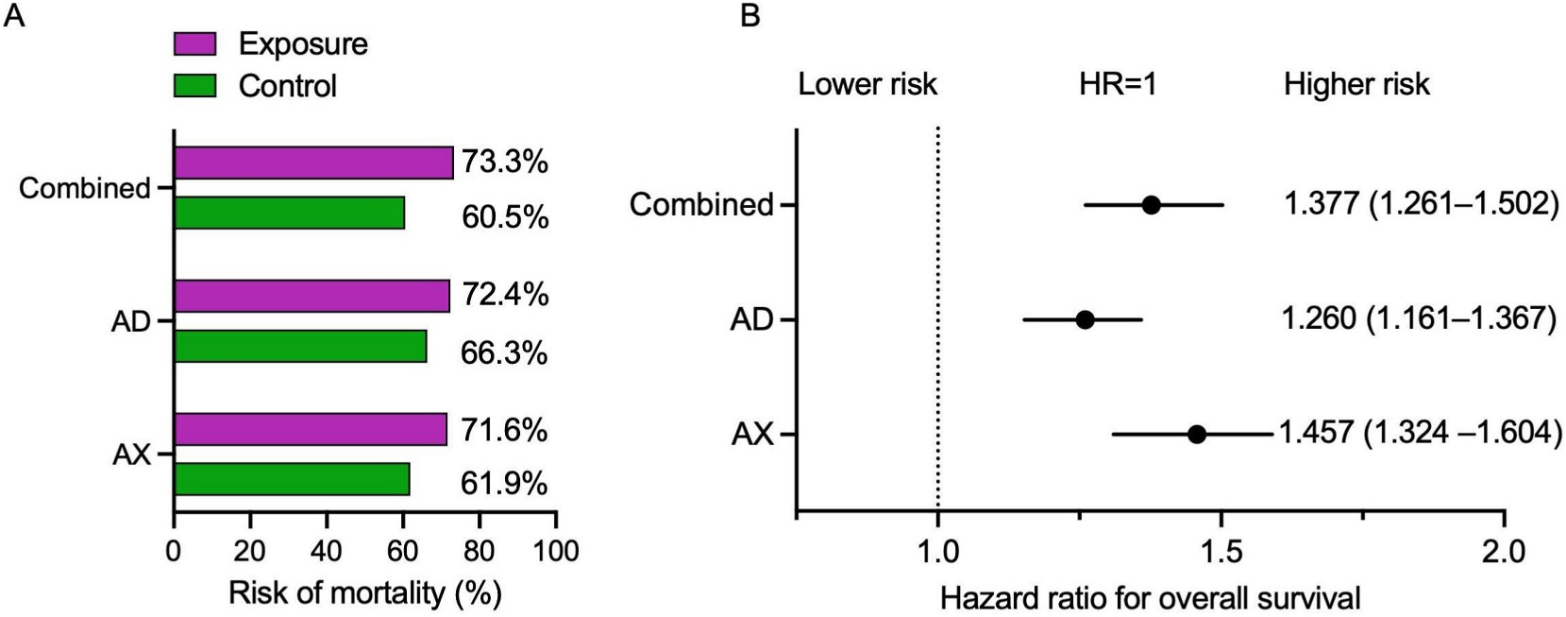
Summary of hazard ratios for overall survival according outcomes according to postoperative psychiatric medication exposure after propensity score matching. (A) Risk of mortality in the exposed and matched control groups across the combined, antidepressant‐only (AD), and anxiolytic‐only (AX) analyses. (B) Forest plot summarising hazard ratios (HR) and 95% confidence intervals for overall survival across the exposure analyses. The dashed vertical line indicates the null value (HR = 1.0).

Across all analyses, postoperative psychiatric medication exposure was consistently associated with poorer OS following RC. Exposure to either antidepressants or anxiolytics was associated with shorter OS than that observed in the corresponding matched control cohorts. In subgroup analyses, the anxiolytic‐only cohort demonstrated the strongest association with poorer OS, exhibiting the highest HR and shortest median OS, followed by the combined exposure and antidepressant‐only analyses (Figure 4).

## 4 Discussion

In this retrospective propensity score–matched cohort study using the TriNetX US Collaborative Network, postoperative psychiatric medication exposure was consistently associated with poorer survival outcomes among patients undergoing RC for bladder cancer. Across both the primary analysis and the medication‐specific subgroup analyses, patients who initiated antidepressants and/or anxiolytics after surgery experienced higher mortality rates and significantly shorter OS than matched controls without psychiatric medication exposure. Notably, the strongest associations were observed in the anxiolytic‐only subgroup, which demonstrated the highest HR and shortest median OS. Collectively, these findings suggest that postoperative psychiatric medication use may serve as a clinically meaningful real‐world marker of psychological burden and survivorship vulnerability following RC. Our results extend prior observations linking psychiatric morbidity to adverse outcomes in patients with bladder cancer and highlight the potential prognostic relevance of postoperative mental health challenges in this population [13, 20].

The clinical relevance of these findings should be interpreted within the unique survivorship context of MIBC. Although RC remains the standard curative treatment for eligible patients with localised disease, the procedure is frequently associated with substantial postoperative physiological, functional, and psychosocial challenges [21, 22]. Urinary diversion, altered body image, sexual dysfunction, chronic fatigue, fear of recurrence, and difficulties with long‐term social and lifestyle adaptation can profoundly influence quality of life and psychological well‐being following RC. Prior survivorship studies have shown that RC adversely affects urinary, bowel, sexual and psychosocial functioning, with persistent impairments in body image, sexual function and psychological distress observed in a substantial proportion of patients undergoing urinary diversion [23, 24]. Within this context, rather than reflecting medication effects alone, postoperative psychiatric medication exposure may identify patients with greater postoperative supportive care needs and psychological vulnerability following RC. This interpretation is particularly relevant because psychosocial burden is often under‐recognised and incompletely documented in routine oncology practice and structured electronic health records. Consequently, psychiatric medication exposure may offer a pragmatic and readily identifiable real‐world indicator of postoperative psychological burden among patients recovering from RC [5, 7].

Our findings are broadly consistent with the psycho‐oncology literature demonstrating that psychiatric morbidity is associated with adverse outcomes in patients with cancer. Depression, anxiety and psychological distress have been linked to poorer quality of life, reduced treatment adherence, increased healthcare utilisation, and less favourable survivorship outcomes across diverse malignancies. Meta‐analyses have also reported associations between depressive symptoms and increased mortality among patients with cancer, reinforcing the clinical importance of psychological assessment and support as integral components of oncology care [25, 26]. More recently, a bladder cancer–specific systematic review highlighted the high prevalence of depression, anxiety, sleep disturbances and unmet psychosocial needs in this population, while emphasising the potential benefits of psychiatric interventions and supportive care strategies [27]. Within this broader context, our study extends the existing evidence by demonstrating an association between postoperative antidepressant and anxiolytic exposure and poorer survival outcomes following RC in a disease‐specific surgical cohort. Previous population‐based studies in bladder cancer have demonstrated that psychiatric morbidity and newly diagnosed mental health disorders are associated with worse overall and cancer‐specific survival [22]. However, most prior investigations have focussed on formally diagnosed psychiatric disorders or have included heterogeneous cancer populations, limiting their applicability to the postoperative RC setting. By contrast, the present study evaluated postoperative psychiatric medication exposure as a clinically observable and routinely recorded marker of psychological burden. This approach offers a complementary perspective to diagnosis‐based analyses and may better reflect the real‐world identification and management of psychological distress in clinical practice. For example, Jazzar et al. reported an association between psychiatric illness and poorer survival among patients with bladder cancer, while the CISTO study comprehensively characterised emotional functioning, depression, anxiety and quality‐of‐life outcomes in patients undergoing bladder cancer treatment. Building upon these observations, our study specifically examined the prognostic implications of postoperative antidepressant and anxiolytic use following RC for MIBC [13, 21]. By leveraging routinely captured medication data, this approach provides a pragmatic framework for identifying a potentially vulnerable subgroup of survivors and broadens the scope of psycho‐oncology literature beyond analyses based solely on formal psychiatric diagnosis.

Several mechanisms may plausibly underlie the observed association between postoperative psychiatric medication exposure and adverse survival outcomes. From a behavioural perspective, psychological distress may adversely affect treatment adherence, healthcare engagement, participation in rehabilitation, nutritional status, sleep quality and long‐term self‐management during survivorship [28]. These factors may be particularly relevant following RC, as recovery often requires sustained adaptation to urinary diversion and substantial lifestyle and functional adjustments. From a biological perspective, evidence from psychoneuroimmunology suggests that chronic stress signalling, hypothalamic–pituitary–adrenal (HPA) axis dysregulation, systemic inflammation and alterations in immune function may influence tumour progression and survivorship trajectories [15, 29]. However, the present study was not designed to establish causal mechanistic relationships, and these pathways should therefore be interpreted as biologically plausible explanatory frameworks rather than direct evidence of causation.

Psychiatric medication exposure may reflect underlying psychological burden, behavioural vulnerability or broader clinical complexity rather than medication‐related biological effects per se. Notably, the anxiolytic‐only subgroup exhibited the strongest association with adverse survival outcomes. Several behavioural, clinical and methodological explanations may plausibly explain this finding [30]. First, postoperative anxiolytic use may identify patients experiencing more severe anxiety, sleep disturbance, panic‐related manifestations or heightened psychological distress during recovery [9, 31]. Second, anxiolytic prescribing may be more common among patients with greater symptom burden, frailty or healthcare complexity, factors that may not be fully captured by the available clinical covariates. In oncology practice, psychotropic medications are often prescribed in response to a constellation of concerns, including sleep difficulties, symptom burden, supportive care needs and functional decline, rather than anxiety symptoms alone [32]. Consequently, anxiolytic exposure may serve as a marker of broader postoperative vulnerability rather than a specific indicator of anxiety [33, 34]. Because residual confounding cannot be completely excluded in observational database research, these subgroup findings should be interpreted cautiously and warrant further prospective studies incorporating validated psychiatric symptom assessment and medication‐specific exposure characterisation [35].

This study has several strengths. First, it leveraged a large, multi‐institutional real‐world electronic health record database, enhancing the generalisability of the findings across diverse healthcare settings and routine clinical practice. Second, PSM was used to minimise baseline imbalance between exposure groups and improve comparability across demographic, clinical and tumour‐related characteristics. Third, the study focussed specifically on postoperative psychiatric medication exposure within a bladder cancer surgical cohort undergoing RC, a clinically important yet relatively underexplored survivorship setting. Finally, separate antidepressant‐only and anxiolytic‐only subgroup analyses enabled evaluation of exposure‐specific patterns beyond the primary combined exposure analysis.

### 4.1 Clinical Implications

Routine recognition of patients initiating postoperative antidepressant or anxiolytic therapy may help identify individuals who could benefit from additional psycho‐oncological assessment and multidisciplinary survivorship care following RC. These findings support the integration of psychosocial evaluation and multidisciplinary survivorship care into the long‐term management of patients with MIBC.

### 4.2 Limitations

Several limitations merit consideration. First, the retrospective observational design precludes causal inference. Second, residual confounding may persist despite PSM, including confounding by indication related to psychiatric medication prescribing. Third, psychiatric medication exposure should not be equated with a confirmed psychiatric diagnosis. Information regarding symptom severity, psychosocial assessments and validated mental health measures was unavailable. Fourth, data regarding medication adherence, treatment duration, dose intensity and specific pharmacologic subclasses were limited, restricting interpretation of medication‐specific effects. Fifth, although oncology‐related covariates were incorporated into the matching process when available, granular information on recurrence status, pathological features, performance status and longitudinal treatment trajectories remained incomplete. Sixth, there is a potential for immortal time bias. Because patients classified as exposed were required to survive long enough to receive at least three postoperative psychiatric medication prescriptions, a guaranteed survival period before fulfilment of the exposure definition was inherent to the study design. Although this exposure definition was selected to improve exposure specificity by reducing isolated or transient prescribing, immortal time bias cannot be completely excluded and should be considered when interpreting the findings. Finally, psychiatric medication exposure may reflect broader postoperative vulnerability, evolving healthcare complexity or overall health status rather than a direct biological determinant of oncologic outcomes. Because of the extended follow‐up inherent to real‐world database studies, medication initiation during survivorship may also be influenced by cancer recurrence, accumulated comorbidities, late‐onset psychological distress or non‐cancer‐related conditions. Although patients receiving antidepressants or anxiolytics before RC were excluded to better identify postoperative medication initiation, untreated or unrecognised pre‐existing psychiatric disorders may still have been present, and confounding from these conditions cannot be completely excluded. Therefore, the observed association between postoperative psychiatric medication exposure and long‐term oncologic outcomes should be interpreted cautiously.

## 5 Conclusion

In this large retrospective propensity score–matched cohort study, postoperative psychiatric medication exposure following RC was associated with poorer OS and increased mortality among patients with bladder cancer. These findings suggest that postoperative psychiatric medication use may serve as a pragmatic real‐world marker of psychological burden and survivorship vulnerability in this population. Future prospective studies incorporating standardised psychiatric assessment, validated measures of symptom severity, targeted survivorship interventions and longitudinal oncological follow‐up are warranted to clarify the underlying mechanisms and to inform multidisciplinary postoperative survivorship care strategies for patients with bladder cancer.

## Author Contributions

**Cheng‐Shuo Huang:** conceptualisation, methodology, formal analysis, investigation, data curation, visualisation, project administration, writing – original draft, writing – review and editing. **Kai‐Hsiang Tung:** conceptualisation, methodology, formal analysis, validation, writing – review and editing. **Ying‐Si Wu:** investigation, validation, writing – review and editing. **Cheng‐Ping Yu:** supervision, resources, writing – review and editing. **Dah‐Shyong Yu:** supervision, resources, writing – review and editing. **Ming‐Hsin Yang:** resources, writing – review and editing. **Yong‐Eng Lee:** resources, data curation, writing – review and editing. **Jar‐Yi Ho:** conceptualisation, supervision, funding acquisition, project administration, writing – review and editing. All authors have read and approved the final manuscript.

## Funding

This work was supported by grants from Tri‐Service General Hospital (TSGH‐E‐113246, TSGH‐114‐248, TSGH‐E‐115248), National Defence Medical University (MNE‐MAB‐E‐113182, MNE‐MAB‐E‐113056, MND‐MAB‐E‐114100, MND‐MAB‐E‐114161, MND‐MAB‐E‐115105, MND‐MAB‐D‐115169), and the National Science and Technology Council, Taiwan (NSTC 114‐2320‐B‐016‐007, NSTC 115‐2320‐B‐016‐010, NSTC 115‐2811‐B‐016‐001 and NSTC 115‐2320‐B‐016 ‐011). Additional support was provided by the National Science and Technology Council Overseas Project for Postgraduate Research (113‐2917‐I‐016‐001 and 113‐2917‐I‐016‐002 to Y.‐S.W. and C.‐S.H.).

## Ethics Statement

This study was approved by the Institutional Review Board of Tri‐Service General Hospital (IRB No. 000513). The requirement for informed consent was waived because the study utilised de‐identified retrospective data from the TriNetX Research Network.

## Supporting information


**Table S1:** Baseline demographic, clinical and tumour characteristics of the antidepressant‐only cohort before and after propensity score matching.

**Table S2:** Baseline demographic, clinical and tumour characteristics of the anxiolytic‐only cohort before and after propensity score matching.

## Data Availability

The data that support the findings of this study were obtained from the TriNetX Research Network. Restrictions apply to the availability of these data, which were used under licence for the current study and are therefore not publicly available. Access to the data may be obtained through the TriNetX Research Network upon reasonable request and in accordance with applicable institutional and data‐use agreements.
