# Supplementary material for: Association of Postoperative Antidepressant and Anxiolytic Exposure With Overall Survival Following Radical Cystectomy for Bladder Cancer
Source: Psychooncology. 2026 Aug 17;35(8):e70581. doi: 10.1002/pon.70581 (PMC13479785; doi:10.1002/pon.70581)
Supplement: Supplementary file 1 [file PON-35-e70581-s001.docx]

Supplementary Table 1. Baseline demographic, clinical and tumour characteristics of the antidepressant-only cohort before and after propensity score matching

| Variable | Before PSM, n (%) | | | After PSM, n (%) | | |
| --- | --- | --- | --- | --- | --- | --- |
|  | **Antidepressant (+) (N = 2,209)** | **Control (N = 10,674)** | **Std Diff** | **Antidepressant (+) (N = 1,682)** | **Control (N = 1,682)** | **Std Diff** |
| Demographics |  |  |  |  |  |  |
| Age, mean ± SD | 69.4 ± 10.8 | 69.0 ± 10.1 | 0.039 | 69.4 ± 10.7 | 69.6 ± 9.9 | 0.013 |
| Female sex | 581 (26.4%) | 2354 (22.3%) | 0.096 | 428 (25.4%) | 422 (25.1%) | 0.008 |
| White race | 1827 (83.0%) | 8338 (79.0%) | 0.105 | 1355 (80.6%) | 1352 (80.4%) | 0.004 |
| Black/African American | 168 (7.6%) | 829 (7.8%) | 0.008 | 139 (8.3%) | 142 (8.4%) | 0.006 |
| Asian | 82 (3.7%) | 523 (5.0%) | 0.06 | 75 (4.5%) | 71 (4.2%) | 0.012 |
| Hispanic/Latino ethnicity | 67 (3.0%) | 365 (3.5%) | 0.023 | 58 (3.4%) | 55 (3.3%) | 0.01 |
| Clinical comorbidities | |  |  |  |  |  |
| Hypertension | 1600 (72.7%) | 5539 (52.4%) | 0.429 | 1224 (72.8%) | 1229 (73.1%) | 0.007 |
| Hyperlipidaemia | 1221 (55.5%) | 3633 (34.4%) | 0.434 | 921 (54.8%) | 943 (56.1%) | 0.026 |
| Diabetes mellitus | 652 (29.6%) | 2150 (20.4%) | 0.216 | 502 (29.8%) | 519 (30.9%) | 0.022 |
| Chronic kidney disease (CKD) | 893 (40.6%) | 1813 (17.2%) | 0.535 | 619 (36.8%) | 591 (35.1%) | 0.035 |
| Obesity / Overweight | 621 (28.2%) | 1523 (14.4%) | 0.342 | 456 (27.1%) | 463 (27.5%) | 0.009 |
| Circulatory diseases | 2000 (90.9%) | 7279 (68.9%) | 0.571 | 1511 (89.8%) | 1509 (89.7%) | 0.004 |
| Genitourinary diseases | 2071 (94.1%) | 7899 (74.8%) | 0.554 | 1570 (93.3%) | 1574 (93.6%) | 0.01 |
| Nervous system disorders | 1623 (73.8%) | 3108 (29.4%) | 0.99 | 1128 (67.1%) | 1141 (67.8%) | 0.016 |
| Mental/behavioural disorders | 1545 (70.2%) | 2872 (27.2%) | 0.954 | 1048 (62.3%) | 1071 (63.7%) | 0.028 |
| Tumour characteristics | |  |  |  |  |  |
| Stage II disease | 184 (8.4%) | 417 (3.9%) | 0.184 | 100 (5.9%) | 88 (5.2%) | 0.031 |
| Stage III disease | 126 (5.7%) | 267 (2.5%) | 0.161 | 74 (4.4%) | 64 (3.8%) | 0.03 |
| Stage IV disease | 55 (2.5%) | 122 (1.2%) | 0.101 | 28 (1.7%) | 32 (1.9%) | 0.018 |
