# Supplementary material for: Association of Postoperative Antidepressant and Anxiolytic Exposure With Overall Survival Following Radical Cystectomy for Bladder Cancer
Source: Psychooncology. 2026 Aug 17;35(8):e70581. doi: 10.1002/pon.70581 (PMC13479785; doi:10.1002/pon.70581)
Supplement: Supplementary file 2 [file PON-35-e70581-s002.docx]

Supplementary Table 2. Baseline demographic, clinical and tumour characteristics of the anxiolytic-only cohort before and after propensity score matching

| Variable | Before PSM, n (%) | | | After PSM, n (%) | | |
| --- | --- | --- | --- | --- | --- | --- |
|  | **Anxiolytic (+) (N = 1,505)** | **Control (N = 11,239)** | **Std Diff** | **Anxiolytic (+) (N = 1,269)** | **Control (N = 1,269)** | **Std Diff** |
| Demographics |  |  |  |  |  |  |
| Age, mean ± SD | 68.1 ± 10.9 | 69.6 ± 9.9 | 0.146 | 68.8 ± 10.5 | 69.1 ± 10.2 | 0.029 |
| Female sex | 442 (29.6%) | 2508 (22.5%) | 0.163 | 349 (27.5%) | 354 (27.9%) | 0.009 |
| Male sex | 1048 (70.2%) | 8629 (77.4%) | 0.164 | 919 (72.4%) | 914 (72.0%) | 0.009 |
| White race | 1239 (83.0%) | 8744 (78.5%) | 0.117 | 1037 (81.7%) | 1046 (82.4%) | 0.018 |
| Black/African American | 102 (6.8%) | 811 (7.3%) | 0.017 | 92 (7.2%) | 89 (7.0%) | 0.009 |
| Asian | 55 (3.7%) | 579 (5.2%) | 0.073 | 54 (4.3%) | 45 (3.5%) | 0.037 |
| Clinical comorbidities | |  |  |  |  |  |
| Hypertension | 1061 (71.1%) | 5796 (52.0%) | 0.401 | 899 (70.8%) | 916 (72.2%) | 0.03 |
| Hyperlipidaemia | 772 (51.7%) | 3896 (35.0%) | 0.344 | 678 (53.4%) | 680 (53.6%) | 0.003 |
| Diabetes mellitus | 432 (29.0%) | 2373 (21.3%) | 0.177 | 381 (30.0%) | 382 (30.1%) | 0.002 |
| Chronic kidney disease (CKD) | 615 (41.2%) | 1922 (17.2%) | 0.546 | 481 (37.9%) | 481 (37.9%) | <0.001 |
| Obesity / Overweight | 416 (27.9%) | 1537 (13.8%) | 0.352 | 346 (27.3%) | 345 (27.2%) | 0.002 |
| Circulatory diseases | 1338 (89.7%) | 7642 (68.6%) | 0.538 | 1125 (88.7%) | 1119 (88.2%) | 0.015 |
| Genitourinary diseases | 1428 (95.7%) | 8107 (72.7%) | 0.664 | 1207 (95.1%) | 1212 (95.5%) | 0.019 |
| Nervous system disorders | 1105 (74.1%) | 3211 (28.8%) | 1.015 | 890 (70.1%) | 886 (69.8%) | 0.007 |
| Mental/behavioural disorders | 1042 (69.8%) | 3080 (27.6%) | 0.931 | 829 (65.3%) | 810 (63.8%) | 0.031 |
| Tumour characteristics | |  |  |  |  |  |
| Stage II disease | 73 (4.9%) | 385 (3.5%) | 0.072 | 48 (3.8%) | 47 (3.7%) | 0.004 |
| Stage III disease | 57 (3.8%) | 228 (2.0%) | 0.105 | 38 (3.0%) | 31 (2.4%) | 0.034 |
| Stage IV disease | 41 (2.7%) | 86 (0.8%) | 0.151 | 22 (1.7%) | 24 (1.9%) | 0.012 |
